# Low Risk Perception of Harm From Substance Use and Sexual Behaviors Among Online Help–Seeking Sexual and Gender Minoritized People in San Francisco, California: Cross-Sectional Survey

**DOI:** 10.2196/81753

**Published:** 2025-10-03

**Authors:** Jarett Maycott, Sean Arayasirikul

**Affiliations:** 1 San Francisco Department of Public Health Center for Public Health Research San Francisco, CA United States; 2 The Legacy Center Joe C Wen School of Population & Public Health University of California, Irvine Irvine, CA United States

**Keywords:** substance use, digital health, sexual and gender minoritized community health, social media

## Abstract

**Background:**

Substance use and HIV epidemics have disproportionately affected sexual and gender minoritized (SGM) communities, with heightened risks among men who have sex with men (MSM) and transgender women of color due to intersecting challenges like poverty, mental health issues, and discrimination. Despite overall declines in substance use and sexual risk behaviors in the general population, these issues persist within SGM communities, exacerbated by stigma and systemic barriers to care. Digital health interventions have emerged as promising tools to address these disparities, offering accessible and stigma-reducing alternatives to traditional care, particularly effective among younger individuals and in underserved areas.

**Objective:**

This study seeks to examine the social correlates of substance use and sexual risk perception among an online sample of help-seeking MSM and transgender women in San Francisco, California.

**Methods:**

We recruited 409 help-seeking MSM and transgender women by using social media advertisements on Facebook, Instagram, and Grindr in 2022-2024. Participants provided informed consent and completed a baseline assessment.

**Results:**

Utilization of testing resources for HIV and hepatitis was high among the participants (401/409, 98.04% and 360/409, 88.02%, respectively). Knowledge of HIV or other sexually transmitted infection health services was also high (379/409, 92.67%). Fewer participants (264/409, 64.55%) were knowledgeable about substance use–related services. Although many participants reported that using substances posed a high risk of harm, some perceived engaging in condomless sex, using prescription opioid drugs without a prescription, and using substances during sex as low risk (122/409, 29.83%, 41/409, 10.02%, and 60/409, 14.67%, respectively). Participants who reported experiencing unstable housing were more likely to report perceiving sharing needles (adjusted odds ratio [aOR] 7.20, 95% CI 1.99-27.80) and nonprescription opioid use (aOR 4.02, 95% CI 1.08-14.90) as low risk. Participants who reported an income below the federal poverty level were more likely to report perceiving sharing needles (aOR 6.35, 95% CI 1.84-23.40), prescription opioid use (aOR 2.89, 95% CI 1.32-6.18), and substance use during sex (aOR 2.29, 95% CI 1.14-4.48) as low risk. Participants who have not been tested for hepatitis in the past have 3.31 times the odds of perceiving prescription opioid use as low risk compared to counterparts who have been tested for hepatitis before (95% CI 1.36-7.68).

**Conclusions:**

This study underscores the importance of social determinants in shaping low risk perception of the harm associated with substance use behaviors among online help–seeking SGM people in San Francisco. These systemic inequities structure participants’ perceptions, access, and utilization of preventive and public health services. Our findings identify critical opportunities for outreach and preventative efforts needed to serve vulnerable populations.

## Introduction

Substance use and HIV epidemics have disproportionately impacted sexual and gender minoritized (SGM) communities. The burden of substance use within SGM communities is disproportionately high and has increased precipitously during the COVID-19 pandemic [1]. Although there have been decreases in illicit substance use and sexual risk behaviors among the general population, associations between the two have persisted among SGM communities [1]. Men who have sex with men (MSM) of color and transgender women of color face heightened vulnerability to concurrent epidemics of poverty, poor mental health, discrimination, and substance use, each constituting a unique barrier to engagement in health care that perpetuates a cycle of neglect [2]. Researchers investigating factors associated with substance use and HIV found that Black MSM experienced an 8% decrease in the odds of reporting viral suppression for every unit increase in the substance abuse and mental illness symptoms [3]. A study in Massachusetts found a higher prevalence of homonegativity among MSM of color, suggesting that internalized stigma may be racialized [1]. Research on adherence to HIV treatment describes how HIV-related stigma and substance use stigma drive poor HIV care outcomes [4,5].

The confluence of these factors has created a unique environment for digital health interventions to become a vital tool for an evolving public health landscape. Digital health interventions may better serve SGM communities faced with complex decisions that limit their access to essential health services [6,7]. For example, researchers issued a self-administered survey to students and faculty in Oklahoma, Wyoming, and Texas, asking whether respondents had ever needed to visit a health care provider but did not in the last 6 months [8]. Their analysis found that SGM respondents experienced a higher likelihood of forgoing care compared to their non-SGM counterparts, suggesting that SGM communities disproportionately experience unmet health care needs [8]. Digital health interventions have been effective in addressing poor mental health among people living with HIV, especially for younger participants [9]. Digital health interventions have demonstrated potential in overcoming barriers rooted in complex stigma and isolation and mitigate stigma as a barrier to accessing health care [9].

Not only that but digital health interventions can also reach SGM populations where they are using online methods and having greater presence in online community spaces [10]. The AFFIRM study delivered cognitive behavioral therapy by using virtual conference technology to overcome traditional in-person barriers and was effective in improving the mental health of SGM youth [11]. Social networking apps have become digital spaces that lead to real-world connections for SGM communities. This can include strengthening community ties, forming relationships, and seeking sexual partners. Yet, these digital spaces can also facilitate sexual and substance use risk behaviors [12,13]. Studies have found that risk behaviors vary for SGM communities depending on the digital space or virtual platform [14,15]. This study seeks to examine the social correlates of substance use and sexual risk perception in an online sample of help-seeking MSM and transgender women in San Francisco, California. These data may inform public health strategies and future interventions to serve SGM communities at risk of and desiring help for substance use and HIV-related services.

## Methods

### Ethical Considerations

All procedures were in accordance with the ethical standards of the institutional and/or national research committee and with the 1964 Helsinki Declaration and its later amendments or comparable ethical standards. The study protocol was approved by the institutional review board of the University of California, San Francisco (approval 20-33169) and covered secondary analyses without additional consent. Participants provided informed consent and were remunerated US $30 for completing the baseline assessment. Data for this secondary analysis were deidentified to protect participants’ privacy and confidentiality.

### Brief Description of the Parent Study

Health eNavigation 2.0 (or Health eNav 2.0) is a short, brief digital health intervention that connects participants to their own digital navigator through SMS text messaging to achieve behavior changes related to access and utilization of substance use and HIV prevention and treatment services. Participants were included in this study if they were interested in seeking help for substance use prevention or treatment and other related topics (eg, HIV/AIDS, mental health), were aged 18 years or older, resided in San Francisco, identified as a cisgender man who had sex with men or a transgender woman, were English-speaking, and had access to a smartphone. The digital navigator provided social support, motivational interviewing [16], and resource navigation and referrals to support participants’ behavior change goals. The theoretical framework for this intervention has been described previously and applied to a sample of young people living with HIV to improve HIV care engagement [17].

### Study Design and Recruitment

We recruited 409 participants online by using social media advertisements on Facebook, Instagram, and Grindr in 2022-2024. Advertisements sought out potential participants looking for help for substance use and interested in participating in the Health eNav 2.0 study. Interested potential participants clicked on the advertisement and were directed to a study interest website where they provided their contact information to study staff, and a staff person contacted them to schedule a remote enrollment visit via Zoom. During the enrollment visit, participants were screened for eligibility, and they provided informed consent electronically by using DocuSign. The enrolled participants completed a baseline assessment. They met with their digital navigator to cocreate a brief service plan consisting of specific goals, objectives, and action steps related to their interest in the project. For example, a participant may be interested in using methamphetamine less. In conversation with their digital navigator, the participant may identify the consequences of their use, the reasons for their use, and actionable steps toward accessing treatment. After completion of the brief service plan, the digital navigator instructed participants on how to set up and use a password-protected, Health Insurance Portability and Accountability Act (HIPAA)-compliant text messaging platform. For the next 30 days, participants worked with their digital navigator over text messaging to enact their brief service plan. The digital navigator used motivational interviewing and social support to elicit change talk with participants during the intervention period. Participants completed an exit assessment at 1 month and a follow-up assessment at 3 months following exit.

### Data Collection and Measures

#### Demographics

We measured age, race/ethnicity, socioeconomic status, housing stability, gender, and sexual orientation. Participants were asked to self-identify their race/ethnicity. Participants who indicated 2 or more races/ethnicities were coded as multiracial. Socioeconomic status was measured using self-reported categories of household income in the previous year. Categories included US $0-$10,000, US $10,001-$30,000, US $30,001-$50,000, US $50,001-$70,000, and higher than US $70,000. Using the 2022 federal poverty line, participants who reported US $10,000 or less were coded as below the federal poverty line [18]. Participants were asked to describe their housing situation. We measured housing as a dichotomous variable where participants who reported living in their own home or apartment was coded as having stable housing. Unstable housing included the following responses: living in a relative’s home, in a group home, in a campus/dormitory housing, in a foster home, in a homeless shelter, or other. Participants self-reported their gender identity as cisgender man or transgender woman or other. Sexual orientation was measured with a self-reported question asking participants to select a response that best fit their sexual orientation between the following: gay/lesbian, bisexual, heterosexual or straight, and queer, pansexual, questioning, and/or other.

#### Knowledge and Utilization of Testing and Preventive and Treatment Services

The history of HIV testing was measured by asking participants, “Have you ever been informed of your HIV status (that is, whether or not you are HIV-positive) based on the result of an HIV test?” History of viral hepatitis testing was measured by asking participants, “Have you ever been informed of your viral hepatitis status (that means whether or not you are infected with the hepatitis virus) based on the result of a viral hepatitis test?” Participants’ knowledge of HIV prevention and treatment services was assessed by asking, “Would you know where to go near where you live to see a health care professional regarding HIV/AIDS or other sexually transmitted health issues?” Participants’ knowledge of substance use prevention and treatment services was assessed by asking, “Would you know where to go near where you live to see a health care professional regarding a drug or alcohol problem?” Responses for these items included yes, no, and do not know. We created dichotomous variables (yes/no) for these items, coding do not know as no.

#### Substance Use and Sex-Related Risk Perception

We measured participants’ perception of the risk of people harming themselves when using specific substances and engaging in risky sexual behaviors. We measured participants’ perception of risk related to sharing needles by asking, “What level of risk do you think people have of harming themselves physically if they share needles, syringes, or other injection equipment when using drugs once or twice a week?” This item was repeated to assess participants’ perception of risk related to using nonprescription opioid drugs (eg, heroin, fentanyl), prescription opioid drugs without a prescription (eg, pain relievers), condomless sex, and substance use during sex. For example, participants’ perception of risk related to substance use during sex was measured by asking, “What level of risk do you think people have of harming themselves if they have sex while high on drugs or under the influence of alcohol?” Responses included no risk, slight risk, moderate risk, great risk, and unknown risk. Low risk perception was coded as answering no, slight, or unknown risk. High risk perception was coded as answering moderate or great risk. These items are drawn from the National Survey of Drug Use and Health [19,20] and were adopted by the Substance Abuse and Mental Health Services Administration as reporting requirements for the evaluation of funded projects.

### Statistical Analysis

This study is a cross-sectional analysis of baseline data that included social determinants of health and risk-related perceptions toward substance use behaviors and potential drivers of HIV risk. We used univariate statistics to describe the sample and variables of interest, including independent and dependent variables. Logistic regression was used to identify the social correlates of low risk perception of harm attributed to substance use and HIV-related behaviors. We built logistic regression models for each outcome controlling for age, gender, and sexual orientation. Analyses were performed using STATA software (version 17; STATA Corp, LLC).

## Results

Table 1 describes participants’ demographics, knowledge and utilization of testing and preventive services, and perceptions of harm attributed to substance use behaviors. Almost a third of participants (117/409, 28.61%) were aged 50 years or older, and 27.38% (112/409) were aged 30-39 years. The majority (327/409, 79.95%) identified as a cisgender man, and 20.05% (82/409) identified as a transgender women or other gender. Most participants identified as White or Latino/a/x/e racial/ethnic (169/409, 41.32% and 113/409, 27.63%, respectively). Approximately 17.60% (72/409) of the participants reported having unstable housing, and 17.36% (71/409) reported an annual income below the 2022 federal poverty line. Utilization of testing resources for HIV and hepatitis was high among our participants, with 98.04% (401/409) having previously been tested for HIV and 88.02% (360/409) for hepatitis. Knowledge of HIV or other sexually transmitted infection health services was also high (379/409, 92.67%). However, participants were less knowledgeable about substance use–related services, with 64.55% (264/409) of the participants reporting they were aware of the resources.

**Table 1:** Sociodemographic characteristics, knowledge and utilization of testing and preventive resources, and risk perception among digital help–seeking men who have sex with men and transgender women in San Francisco, California, in 2021-2024 (N=409).

Characteristic				Values, n (%)
**Demographics**				
	**Age (years)**			
		18-29	103 (25.18)	
		30-39	112 (27.38)	
		40-49	77 (18.83)	
		50+	117 (28.61)	
	**Race/ethnicity**			
		White	169 (41.32)	
		Latine	113 (27.63)	
		Asian and Pacific Islander and Native Hawaiian	50 (12.22)	
		Black	38 (9.29)	
		Multiracial/other	39 (9.54)	
	**Gender**			
		Cisgender man	327 (79.95)	
		Transgender woman/other	82 (20.05)	
	**Sexual orientation**			
		Bisexual	47 (11.49)	
		Gay/lesbian	282 (68.95)	
		Other	48 (11.74)	
		Straight/heterosexual	32 (7.82)	
	**Housing stability**			
		Stable	337 (82.40)	
		Unstable	72 (17.60)	
	**Socioeconomic status**			
		Above federal poverty line	338 (82.64)	
		Below federal poverty line	71 (17.36)	
**Knowledge and utilization of testing and preventive and treatment services**				
	**History of HIV testing**			
		No	8 (1.96)	
		Yes	401 (98.04)	
	**History of hepatitis testing**			
		No	49 (11.98)	
		Yes	360 (88.02)	
	**Knowledge of where to access HIV or sexually transmitted infection services**			
		No	30 (7.33)	
		Yes	379 (92.67)	
	**Knowledge of where to access substance use services**			
		No	145 (35.45)	
		Yes	264 (64.55)	
**Substance use and sexual risk perception**				
	**Sharing needles**			
		High risk perception	397 (97.07)	
		Low risk perception	12 (2.93)	
	**Nonprescription opioid drugs**			
		High risk perception	397 (97.07)	
		Low risk perception	12 (2.93)	
	**Prescription opioid drugs without a prescription**			
		High risk perception	368 (89.98)	
		Low risk perception	41 (10.02)	
	**Condomless sex**			
		High risk perception	287 (70.17)	
		Low risk perception	122 (29.83)	
	**Substance use during sex**			
		High risk perception	349 (85.33)	
		Low risk perception	60 (14.67)	

Overall, participants frequently reported that using substances posed high risk of harm. Approximately 97.07% (397/409) of the participants perceived sharing needles and using nonprescription opioid drugs as high risk, followed by prescription opioid drugs without a prescription, substance use during sex, and condomless sex (368/409, 89.98%, 349/409, 85.33%, and 287/409, 70.17%, respectively). Conversely, almost a third (122/409, 29.83%) perceived engaging in condomless sex as low risk. One in ten (41/409, 10.02%) perceived using prescription opioid drugs without a prescription as low risk, and 14.67% (60/409) perceived using substances during sex as low risk.

Table 2 describes the associations between social factors and low risk perception of harm attributed to substance use and HIV risk behaviors. We assessed the following social factors: race, housing stability, and socioeconomic status, history of receiving testing services, and knowledge of substance use and HIV-related services. Odds ratios and 95% CIs are presented, adjusted for age, gender, and sexual orientation.

Participants who reported experiencing unstable housing were more likely to report perceiving sharing needles (adjusted odds ratio [aOR] 7.20, 95% CI 1.99-27.80) and nonprescription opioid use (aOR 4.02, 95% CI 1.08-14.90) as low risk. Participants who reported an income below the federal poverty level were more likely to report perceiving sharing needles (aOR 6.35, 95% CI 1.84-23.40), prescription opioid use (aOR 2.89, 95% CI 1.32-6.18), and substance use during sex (aOR 2.29, 95% CI 1.14-4.48) as low risk. Participants who have not been tested for hepatitis in the past have 3.31 times the odds of perceiving prescription opioid use as low risk compared to counterparts who have been tested for hepatitis before (95% CI 1.36-7.68).

**Table 2:** Social correlates of low risk perception toward substance use and sexual risk among online help–seeking men who have sex with men and transgender women in San Francisco, California, in 2021-2024 (N=409).

Characteristic			Sharing needles, aOR^a^ (95% CI)		Nonprescription opioids, aOR (95% CI)		Prescription opiods, aOR (95% CI)		Condomless sex, aOR (95% CI)		Substance use during sex, aOR (95% CI)
**Race/ethnicity**											
	White	Ref^b^		Ref		Ref		Ref		Ref	
	Latine	3.97 (0.86-28.3)		0.92 (0.21-3.62)		0.74 (0.30-1.70)		0.63 (0.36-1.09)		0.86 (0.42-1.72)	
	Asian, Pacific Islander, and Native Hawaiian	1.90 (0.08-21.9		0.57 (0.03-4.09)		1.32 (0.43-3.62)		1.33 (0.67-2.60)		1.17 (0.47-2.74)	
	Black	1.50 (0.06-17.7)		0		0.55 (0.11-2.01)		0.17 (0.04-0.51)		0.15 (0.01-0.77)	
	Multiracial (non-Latino)/other	4.40 (0.44-44.8)		0.49 (0.02-4.07)		0.80 (0.20-2.55)		0.44 (0.17-1.03)		0.66 (0.20-1.84)	
**Housing stability**											
	Stable	Ref		Ref		Ref		Ref		Ref	
	Unstable	7.20 (1.99-27.8)		4.02 (1.08-14.9)		1.26 (0.52-2.86)		0.69 (0.36-1.28)		1.08 (0.48-2.27)	
**Socioeconomic status**											
	Above federal poverty line	Ref		Ref		Ref		Ref		Ref	
	Below federal poverty line	6.35 (1.84-23.4)		2.14 (0.51-7.77)		2.89 (1.32-6.18)		0.69 (0.36-1.25)		2.29 (1.14-4.48)	
**Has been tested for hepatitis**											
	Yes	Ref		Ref		Ref		Ref		Ref	
	No	2.82 (0.57-10.8)		2.94 (0.58-11.6)		3.31 (1.36-7.68)		1.12 (0.56-2.16)		0.96 (0.38-2.16)	
**Has been tested for HIV**											
	Yes	Ref		Ref		Ref		Ref		Ref	
	No	7.69 (0.36-60.7)		4.75 (0.21-39.4)		0.98 (0.05-6.65)		0.85 (0.12-3.95)		2.12 (0.29-10.5)	
**Knowledge of HIV/sexually transmitted infection services**											
	Yes	Ref		Ref		Ref		Ref		Ref	
	No	1.43 (0.08-8.27)		1.41 (0.07-8.71)		0.63 (0.10-2.31)		0.58 (0.21-1.39)		0.96 (0.27-2.66)	
**Knowledge of substance use–related services**											
	Yes	Ref		Ref		Ref		Ref		Ref	
	No	1.36 (0.38-4.56)		1.24 (0.31-4.29)		0.65 (0.29-1.36)		0.52 (0.32-0.84)		0.72 (0.38-1.30)	

^a^aOR: adjusted odds ratio.

^b^Ref: reference.

## Discussion

This study examines how SGM people seeking help for substance use–related services online perceive substance use risk and harms. We found that the highest proportion of participants perceived sharing needles and nonprescription opioid use as high risk, followed by prescription opioid drugs without a prescription, substance use during sex, and condomless sex. On the surface, these data demonstrate that participants are actively engaged in assessing risk and harms related to using specific substances and appropriately ascribe high risk to substances that have severe health consequences. For example, needle sharing is a mode of transmission for HIV acquisition [21], and use of nonprescription opioids like fentanyl could lead to life-threatening overdose [22]. Yet, for other behaviors like prescription opioid drug use without a prescription, condomless sex, and substance use during sex, more participants perceive these behaviors as less risky.

Our study finds that low risk perception is unevenly distributed, revealing that some people are more likely to perceive substance use behaviors as low risk. For example, although we found that while more than 95% of the participants perceived sharing needles as high risk, participants experiencing unstable housing and those with an income less than the federal poverty level are more likely to perceive sharing needles as low risk compared to their respective counterparts. Similarly, while most participants perceived nonprescription opioid drug use as high risk, people experiencing unstable housing were more likely to perceive this behavior as low risk.

These findings can inform how public health sectors ought to tailor interventions to reach communities who perceive substance use behaviors as low risk. We found that participants who reported an income below the federal poverty level were more likely to report perceiving prescription opioid use as low risk. Integrating substance use prevention and health education into employment programs and social welfare and other benefit programs may improve risk assessment and decision-making skills. For example, Bosk et al [23] describe opportunities for child welfare programs to integrate addiction treatment, education, and prevention among parents of families involved in the child welfare system. Additionally, we found that participants who have not been tested for hepatitis in the past are more likely to perceive prescription opioid use as low risk. Scaling up and enhancing viral hepatitis testing could act as a feasible conduit for health education about substance use disorder and prescription opioid use.

Our study is not without limitations. There is limited generalizability of our findings due to the cross-sectional study design and the unique sociohistorical environment and communities of the San Francisco Bay Area and its public health institutions. Although existing scientific literature suggests that social factors may influence substance use behaviors [24-26], our study cannot definitively establish causality or determine the direction of influence between these social factors and risk perception [27]. Historically, San Francisco has been a center of civil rights and public health movements for SGM communities—from HIV to harm reduction to transgender health to ending the syndemic of hepatitis C virus/HIV/sexually transmitted infections. As a result, not only have many SGM folks relocated to San Francisco to live in an environment less adverse to SGM people, the public health infrastructure has evolved to specifically meet the needs of SGM communities [28]. Despite these limitations, this study is unique in that the sample is comprised of SGM individuals seeking help online. Stigma can deter people from seeking help for substance use, mental health, and HIV services in-person [29], and creating entry points into the public health service system that interface with people seeking help online is critical to overcoming stigma and communities hardly reached by traditional methods [30]. Another limitation to our study may include how response categories were recoded to characterize low risk perception. Although many participants in our study reported knowledge of HIV/sexually transmitted infection services, more than a third were unaware of substance use services; reaching these individuals is critical to addressing gaps in substance use disorder service utilization, and increasing research find that it is important to reach people who use substances online [31,32]. Future research examining pre-exposure prophylaxis for HIV and sexually transmitted infections may be important factors in assessing sexual risk and vulnerability for people who use substances. Although our study examines perceptions of risk of harm to oneself related to substance use quantitatively, we understand that the dynamic processes of cognition and risk assessment in the lived experiences of vulnerable communities may perhaps be best investigated by employing qualitative methodologies. For example, phenomenology and repeated in-depth interviews over time may reveal critical insights into the lived experiences and meaning behind understanding risks related to substance use and decision-making.

This study underscores the importance of social determinants in shaping the low risk perception of harm associated with substance use behaviors among online help–seeking SGM people in San Francisco. These systemic inequities structure participants’ perceptions, access, and utilization of preventive and public health services. Our findings identify critical opportunities for outreach and preventative efforts needed to serve vulnerable populations. Addressing the substance use and HIV syndemic requires public health interventions that seek to overcome structural barriers and leverage digital technology to meet people where they are. Although our study provides valuable insights, further research is needed to understand the influence of social determinants in digital spaces and facilitate the development of effective and inclusive interventions in the future.

## Abbreviations

aOR: adjusted odds ratio
HIPAA: Health Insurance Portability and Accountability Act
MSM: men who have sex with men
SGM: sexual and gender minoritized

## Data Availability

The datasets generated or analyzed during this study are not publicly available as they contain details that could be used to identify participants but are available from the corresponding author on reasonable request.
